# Single-Cell RNA-Seq Analysis Reveals Macrophages Are Involved in the Pathogenesis of Human Sporadic Acute Type A Aortic Dissection

**DOI:** 10.3390/biom13020399

**Published:** 2023-02-20

**Authors:** Bin Zhang, Kuan Zeng, Rui-Cong Guan, Hui-Qi Jiang, Yong-Jia Qiang, Qing Zhang, Mo Yang, Bao-Ping Deng, Yan-Qi Yang

**Affiliations:** 1 Department of Cardiovascular Surgery, Sun Yat-sen Memorial Hospital, Sun Yat-sen University, Guangzhou 510120, China; 2 Guangdong Provincial Key Laboratory of Malignant Tumor Epigenetics and Gene Regulation, Sun Yat-sen Memorial Hospital, Sun Yat-sen University, Guangzhou 510120, China; 3 State Key Laboratory of Biocontrol, School of Life Sciences, Sun Yat-sen University, Guangzhou 528406, China; 4 Scientific Research Center, The Seventh Affiliated Hospital, Sun Yat-sen University, Shenzhen 518107, China; 5 Department of Cardiovascular Surgery, The Fifth Affiliated Hospital of Southern Medical University, Guangzhou 510920, China; 6 Department of Cardiothoracic Surgery, University Hospital, University Linköping, 58183 Linköping, Sweden

**Keywords:** single-cell RNA sequencing, acute type A aortic dissection, macrophage, inflammation, matrix metalloproteinase, MMP2, MMP9

## Abstract

Macrophages play an important role in the progression of sporadic acute type A aortic dissection (ATAAD). The aim of this study was to characterize the cellular heterogeneity of macrophages in ATAAD tissues by scRNA-seq. Ascending aortic wall tissue from six ATAAD patients and three heart transplant donors was assessed by scRNA-seq and then analyzed and validated by various bioinformatic algorithms and histopathology experiments. The results revealed that the proportion of macrophages in ATAAD tissues (24.51%) was significantly higher than that in normal tissues (13.69%). Among the six macrophage subclusters, pro-inflammatory macrophages accounted for 14.96% of macrophages in the AD group and 0.18% in the normal group. Chemokine- and inflammation-related genes (CCL2, CCL20, S100A8, and S100A9) were expressed more intensively in macrophages in ATAAD tissue than in those in normal tissue. Additionally, intercellular communication analysis and transcription factor analysis indicated the activation of inflammation and degradation of the extracellular matrix in ATAAD tissue. Finally, immunohistochemistry, immunofluorescence, and Western blot experiments confirmed the overexpression of macrophage marker genes (CD68 and CD163) and matrix metalloproteinases (MMP9 and MMP2) in ATAAD tissue. Collectively, our study provides a preliminary evaluation of the role of macrophages in ATAAD, and the results could aid in the development of therapeutic options in the future.

## 1. Introduction

Acute type A aortic dissection (ATAAD) is a life-threatening emergency with a mortality rate of 1% to 2% per hour after onset of symptoms in untreated patients [1]. Even at experienced cardiovascular centers, the surgical mortality rate for ATAAD ranges from 10 to 35% [2]. Sporadic aortic dissection, when distinguished from genetic factors such as Marfan syndrome, accounts for the majority of clinically diagnosed aortic dissection (AD) cases, and its pathogenesis has not been elucidated [3,4]. Studies have found that there is significant macrophage infiltration in the aortic walls of AD patients [5]. Macrophages infiltrating the aortic wall tissue may cause elevated oxidative stress and increased expression of inflammatory factors and matrix metalloproteinases (MMPs), thereby promoting vascular smooth muscle cell (VSMC) apoptosis and aortic remodeling, which are factors that play important roles in the pathogenesis of AD [6,7]. Although the study by Liu et al. analyzed the function of immune cells in AD tissues, the study did not subject macrophages to further detailed subpopulation identification or analysis of differential macrophage genes in different tissues [7]. Therefore, it is crucial to study the role of macrophages in the pathogenesis of AD.

Advances in single-cell RNA sequencing (scRNA-seq) have enabled an in-depth understanding of tissue and cell heterogeneity, and precise studies of the roles of single cells, such as macrophages, in AD progression have been conducted [8,9]. Kalluri et al. were the first to report the single-cell atlas of all cells in the mouse aorta. They established that the scRNA-seq approach is more effective for characterizing the cellular heterogeneity of the aorta than conventional methods [10]. Subsequently, Luo et al. found in a mouse model of AD that DNA leaked into the cytoplasm due to nuclear and mitochondrial DNA damage, and the subsequent leak of DNA to the cytosol activated STING signaling, which induced cell death through apoptosis and necroptosis [11]. In a scRNA-seq study of the human aortic wall, Li et al. revealed dynamic cell populations and differential gene expression patterns in control and aneurysmal human aortic tissues [12]. Recent scRNA-seq studies have reported the contribution of immune cells in human AD tissue [7]. However, few scRNA-seq studies have explored the role of macrophages in the formation of ATAAD.

In this study, we characterized the cellular heterogeneity of macrophages in ascending aortic wall tissue from six ATAAD patients and three heart transplant donors by performing scRNA-seq. To comprehensively study the function of macrophages in AD tissue, we performed a subcluster identification analysis, a pseudotime trajectory analysis, a differential gene expression analysis, a cell–cell communication analysis, and a transcription factor analysis on macrophages. In addition, our study confirmed the expression of macrophage marker genes and MMPs in aortic tissue by immunohistochemistry, immunofluorescence, and protein blotting experiments. Overall, our study provides a preliminary evaluation of the role of macrophages in ATAAD, and the results could aid in the development of therapeutic options in the future.

## 2. Materials and Methods

### 2.1. Ethical Approval and Consent

The subject design and the methods used in this study were reviewed and approved by the Research Ethics Committee of Sun Yat-Sen Memorial Hospital, Sun Yat-Sen University (Guangzhou, China). All samples were obtained from deceased transplant organ donors after obtaining Research Ethics Committee approval (Application Number: SYSEC-KY-KS-2020-192). Informed consent was obtained from the subjects’ families.

### 2.2. Acquisition of Donor Samples

The ascending aortic wall samples for this study were obtained from the department of cardiovascular surgery and the Organ Transplantation Center of Sun Yat-Sen Memorial Hospital, Sun Yat-Sen University in accordance with the national organ donation procedure. The ascending aorta tissue (2 × 2 cm) was immersed in GEXSCOPE Tissue Preservation Solution (93774, Singleron Biotechnologies, Cologne, Germany) immediately after acquisition. The samples were stored and transported at 4 °C for preparation of the single-cell suspension. Donors with preoperative diagnoses, including aortic disease (aortic dissection, aortic aneurysm, aortitis, etc.), cardiovascular malformation, connective tissue disease, immune system disease, and malignancy, were excluded.

### 2.3. Preparation of Single-Cell Samples from the Donor Samples

The fresh ascending aortic wall tissues were washed at least three times with Hanks Balanced Salt Solution (HBSS) (14025092, Thermo Fisher Scientific, Waltham, MA, USA) to wash away the blood. The adipose tissue attached to the adventitia was removed by blunt separation using forceps. Single cell preparation was performed on ice to preserve cell viability. The aortic tissues were cut into smaller pieces (1–2 mm^3^) and digested in a 15 mL centrifuge tube with a digestive enzyme cocktail that included 3 mg/mL of Type I collagenase (LS004176, Worthington Biochemical Corp., Lakewood, NJ, USA), 0.156 mg/mL of Type XI collagenase (H3506, Sigma Corp., Kanagawa, Japan), 0.25 mg/mL of soybean trypsin inhibitor (LS003571, Worthington, Singapore), 0.1875 mg/mL of lyophilized elastase (LS002292, Worthington), 0.24 mg/mL of Type I hyaluronidase (H3506, Sigma, St. Louis, MO, USA), and 60 U/mL of DNase I (10104159001, Roche, Shanghai, China), as previously described [12]. The samples were shaken slowly for 2–3 h in a 37 °C water bath to facilitate complete digestion. The digested supernatant was separated from the tissue remnants. The digestive reaction was stopped by adding Dulbecco’s modified Eagle’s medium (DMEM) and 10% fetal bovine serum. The remaining tissue was further digested with a fresh enzyme cocktail. The digestion process was repeated three times to ensure that all cells from the aortic tissues were isolated. The cells were pelleted by centrifugation at 300× *g* and 4 °C for 5 min and resuspended in 1X PBS to generate a single-cell suspension. The cells were counted using the TC20 automated cell counter (Singleron). The live cell counts were obtained by staining with trypan blue (302643, Sigma). Samples with >80% cell viability and 1 × 10^6^ cells/mL were further processed for sequencing.

### 2.4. Single-Cell RNA Sequencing

Single-cell suspensions were processed on the 10× Chromium Single Cell platform in accordance with the manufacturer’s instructions using the 10× Genomics Chromium Single-Cell 3′ kit (V3). The cDNA amplification and library construction steps were performed in accordance with the standard protocol. Single cells with specific 10× Barcode Gel Beads and unique molecular identifiers were partitioned into the Gel Bead-in-Emulsion (GEM) in the GemCode instrument. Then, cell lysis was performed followed by barcoded reverse transcription of the RNA samples, amplification, shearing, attachment of the 5′ adaptors, and sample indexing. Then, the libraries were sequenced using the Illumina NovaSeq 6000 sequencing system (paired-end multiplexing run, 150 bp) by LC-Bio Technology Co., Ltd. (Hangzhou, China).

### 2.5. Single-Cell RNA-Seq Data Processing

Postprocessing and quality control analyses were performed using the 10× Cell Ranger package (v1.2.0:10× Genomics). In the quality control analysis, low-quality cells with >25% reads mapped to the mitochondria or cells with <700 or >15,000 UMI counts were removed. The Doublet Finder R package was then used to remove the doublets. The sequencing data were analyzed using Cell Ranger software (10× Genomics), and the gene expression data of individual cells were extracted. Cell Ranger (http://support.10xgenomics.com/single-cell/software/overview/welcome (accessed on 20 August 2022)) uses an aligner called STAR (https://github.com/alexdobin/STAR (accessed on 20 August 2022)) [13] to perform splice-aware alignment of the RNA-seq reads to the genome. The data were then normalized and converted with a scale factor (the default setting was 10,000) and log-transformed using the Seurat embedded function. The RunPCA function of the Seurat package (v4.0.5) was used to perform the correlation analysis. Then, an integrated analysis of the three datasets was performed as described previously [14].

### 2.6. Dimension–Reduction and Cell Clustering

The top 2000 highly variable genes (HVGs) were extracted from each sample using the FindVariableFeatures function and analyzed by principal component analysis (PCA) [15]. The main cell clusters were identified using the standard Seurat package with a resolution of 0.8. The results with a resolution of 0.6 were chosen for the subclustering analysis of the target cells to demonstrate changes in the subpopulations of all cell types in each sample. Then, the Seurat’s RunUMAP function with dimension parameters (1:30) was used for two-dimensional (2D) visualization.

### 2.7. Differentially Expressed Gene Analysis

The FindMarkers function in Seurat was used to determine the differentially expressed genes (DEGs) in each cell cluster. The Wilcoxon rank-sum test with Bonferroni correction was used to measure the significance of changes in the gene expression between the various subsets of cells. The Benjamini–Hochberg correction method was used for multiple hypothesis correction, and the false discovery rate (FDR) was reflected by the *q*-value [16]. The differentially expressed genes were identified using the following criteria: (1) Wilcoxon rank-sum test *q*-value ≤ 0.01; (2) log2FC ≥ 0.26, where log2FC refers to the log2 fold change of the average gene expression between two groups or subsets of cells; and (3) detection of the gene in >10% of cells in a specific cell cluster.

### 2.8. Marker Gene Analysis and Cell-Type Annotation

The marker genes of each cell cluster relative to the other cell populations were first screened using the results of the Seurat FindMarkers algorithm (test. use = bimod), as previously described [14]. Then, the marker genes were determined based on their known roles in cell biology, the functions of the differentially expression genes in each cell cluster, and the distance relationship between the cells in the reduced dimensional uniform manifold approximation and projection (UMAP) map. The main clusters were manually annotated based on the canonical marker genes and their known functions, as described in previously published reports. The annotations of ambiguous cell subclusters were temporarily substituted with numbers. The heatmaps, dot plots, and the violin plots for the cell-specific markers were generated using the Seurat DoHeatmap/DotPlot/Vlnplot function.

### 2.9. GO and KEGG Enrichment Analysis

A functional enrichment analysis of the gene ontology (GO) terms and the Kyoto encyclopedia of genes and genomes (KEGG) pathways associated with the DEGs was performed using the clusterProfiler (v3.14.3) R package and hypergeometric distribution [17]. The significantly enriched GO terms and KEGG pathways were identified based on the *p*-adj value < 0.05 as the criterion and were visualized using bar plots.

### 2.10. Pseudotime Trajectory Analysis

The trajectory analysis was performed using the Monocle2 R package (v2.14.0) to determine the gene expression changes related to the cell state transitions in the main cell clusters and subclusters [18]. The DDRTree function and the cell ordering algorithm in the Monocle 2 R package were used for reducing the data dimensions and cell ordering, respectively. The significantly altered genes were then extracted from the top 50 markers within the clusters using the differentialGeneTest (fullModelFormulaStr = Pseudotime) function. A representative heatmap was generated using the plot pseudotime heatmap function. The numcluster was set at four to obtain four modules that included genes with significant expression changes and similar trends based on their pseudotemporal expression patterns.

### 2.11. Cell-to-Cell Communication Analysis

The cell-to-cell communication between various cell types in the aorta samples was evaluated using the Cellphone DB resource based on the well-recognized ligand–receptor pairs from the single-cell transcriptomics (scRNAseq) data [19]. The strengths of the ligand–receptor interactions between the cell types were expressed as an interaction score, defined as the product of the average expression of the ligands in one cell type and the average expression of the receptors in another cell type. The predicted interaction pairs with an adjusted *p*-value < 0.05 and an average log expression >0.1 were considered significant and were visualized using the Circlize and Igraph R packages.

### 2.12. Transcription Factor Analysis

The cell-type-specific transcription factors (TFs) of major cells were identified by the Single Cell Regulatory Network Inference and Clustering (SCENIC) R algorithm, a computational method used for simultaneous gene regulatory network reconstruction and cell-state identification from single-cell RNA-seq data [20]. The identification of transcription factors and cell states was conducted by cis-regulatory analysis. By comparing the gene expression in two different cell populations, the enrichment of predicted targets was assessed, and only significant transcription factors were chosen as candidate transcription factors. A regulatory network based on the co-expression of regulatory factors and targets was constructed using the GENIE3 software package [21].

### 2.13. Immunohistology and Western Blot Analyses

A histopathology analysis was performed to validate the results of the single sequencing analysis. The overall morphology and organization of the aortic tissue and the resident cells was evaluated by hematoxylin–eosin (HE) staining (LA2700-250g; Solarbio, Beijing, China). Victoria Blue staining was used to analyze the distribution of elastic fibers in the aortic tissue samples. Masson’s trichrome (SH012; CNT-BIO) staining was used to detect the collagen fibers. Immunohistochemical staining with antibodies against CD14 (1:200, rabbit monoclonal, GB11254; Servicebio, Wuhan, China), CD68 (1:200, rabbit monoclonal, GB113109; Servicebio, Wuhan, China), CD86 (1:200, rabbit monoclonal, GB13585; Servicebio, Wuhan, China), and CD163 (1:200, rabbit polyclonal, GB113751; Servicebio, Wuhan, China) was used to analyze the monocytes and macrophages. Immunofluorescence (mIF) staining with antibodies against MMP9 (1:1000, rabbit monoclonal, GB11132; Servicebio, Wuhan, China), MMP2 (1:1000, rabbit polyclonal, GB11130; Servicebio, Wuhan, China), CD163 (1:1000, rabbit polyclonal, GB113751; Servicebio, Wuhan, China), and CD68 (1:1000, rabbit monoclonal, GB113109; Servicebio, Wuhan, China) was performed to assess the expression of metalloproteinases and the distribution of macrophages in AD tissue and normal tissue. The nuclei were visualized using diamidino-2-phenylindole (DAPI).

The target tissues and macrophages were stained according to the standard protocols. Briefly, fresh aortic wall tissues were fixed with 4% paraformaldehyde immediately after harvesting. The paraffin-embedded tissues were sectioned (5 µm thick), stained with HE, Victoria Blue, and Masson’s trichrome solutions, and observed under a light microscope. The slides used for immunohistochemical staining were prepared by first incubating the paraffin-embedded sections in an oven at 60 °C for 2 h. The dehydrated sections were then incubated in a high-pressure water bath with 10 mM sodium citrate for antigen retrieval. The endogenous peroxidase activity was blocked by incubating the sections with 3% (*v*/*v*) H_2_O_2_ for 30 min at room temperature. Then, the sections were rinsed thrice each with ddH_2_O and PBS for 5 min. Then, the sections were stained with the primary and secondary antibodies in accordance with the manufacturer’s instructions. Finally, the stained sections were scanned in an automatic pathological section scanner (NanoZoomer S360, Hamamatsu Photonics, Beijing, China), and high-resolution images were captured.

The total protein was extracted from the ascending aortic tissue of ATAAD patients and healthy organ donors, and the protein concentration was determined with the BCA Protein Assay Reagent (23225, Thermo Fisher Scientific, Shanghai, China). The sample loading amount was determined based on the sample concentration to ensure that each sample’s total protein loading amount was 40 µg. The proteins were separated using sodium dodecyl sulfate-polyacrylamide gel electrophoresis and transferred to a polyvinylidene difluoride membrane (Bio-Rad Laboratories, Hercules, CA, USA). After blocking with 5% bovine serum albumin (A1933, Sigma-Aldrich), membranes were incubated at 4 °C overnight with the following primary antibodies: anti-MMP9 (1:1000, rabbit monoclonal, GB11132; Servicebio), anti-MMP2 (1:1000, rabbit polyclonal, GB11130; Servicebio), anti-CD163 (1:1000, rabbit polyclonal, GB113751; Servicebio), anti-CD68 (1:1000, rabbit monoclonal, GB113109; Servicebio), and β-actin (1:1000, rabbit polyclonal, GB111364; Servicebio). Then, the membranes were incubated with horseradish peroxidase (HRP)-conjugated secondary antibodies (goat anti-rabbit IgG, 1:5000, G1213-100UL; Servicebio) after washing. The target proteins were detected with Clarity Western ECL Substrate 1:1 (Bio-Rad Laboratories, USA) and measured using Image Quant LAS 4000 CCD imager and software (GE Healthcare, Life Sciences, Chicago, IL, USA).

### 2.14. Statistical Analysis

The statistical analysis was performed using R package version 4.1.0 (https://www.r-project.org (accessed on 20 August 2022)), GraphPad Prism 8.0 software (GraphPad Software, San Diego, CA, USA), and SPSS software version 20.0 (Chicago, IL, USA). The continuous variables are presented as the mean ± standard deviation (SD). The categorical variables are presented as the number of categories or percentage (%). The statistical significance of the differences between the datasets was evaluated by the ANOVA or the Student’s *t*-test and the Student–Newman–Keuls post hoc test. FDR < 0.05 was considered statistically significant.

## 3. Results

### 3.1. Overview of Single-Cell Transcriptomic Data from AD and Normal Ascending Aorta Tissues

**Figure 1:**
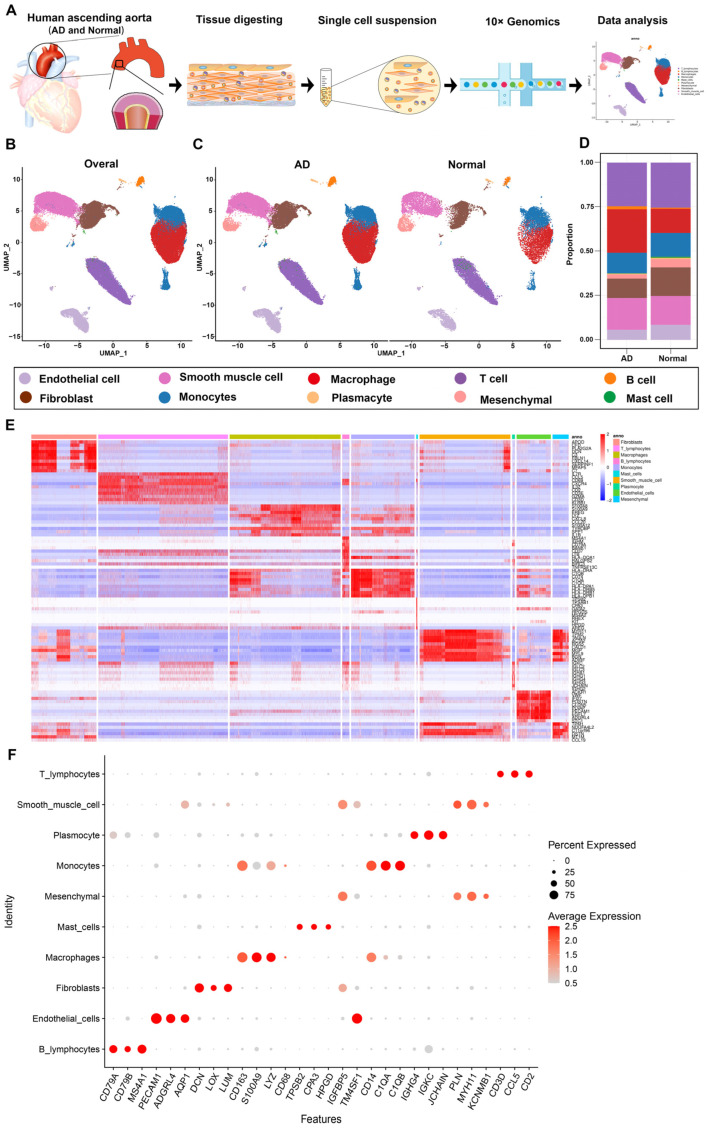
Single-cell atlas of AD and normal ascending aorta samples. (**A**) Schematic diagram of the sample processing and experimental workflow employed. (**B**) UMAP plot of the 79,544 cells profiled, including ten main cell types in nine samples. Below the graph are legends color-coded by the assigned cell type. (**C**) UMAP plots of the AD group (six samples) and normal group (three samples). (**D**) Bar plot depicting the proportions of the ten main cell types in the AD and normal groups. (**E**) Heatmap of the top ten DEGs for the ten main cell types. (**F**) Dot plot of selected marker genes for each cell type. The dot size represents the percentage of cells expressing each gene, while the dot color represents the level of expression. UMAP, uniform manifold approximation and projection; DEGs, differentially expressed genes.

We obtained a total of nine ascending aortic tissue samples from six patients with sporadic ATAAD and three heart transplant donors. The participants’ clinical information is summarized in Table S1. Single-cell suspensions were prepared from the ascending aorta tissues and sequenced on the 10× Genomics platform (Figure 1A). After pre-processing and a quality assessment, 79,544 cells were considered eligible for the final analysis. The unsupervised cell clustering algorithm was used to classify all cells into 10 cell clusters based on known cellular characteristics (Figure 1B). The samples were divided the two groups, namely, an AD group, which included six samples from patients with ATAAD, and a normal group, which included three samples from heart transplant donors. These groups were studied to determine the characteristics of the disease. The UMAP results of the AD and normal groups were consistent with those obtained from the overall analysis (Figure 1C).

The proportion of cells in each group was plotted to eliminate errors in comparison because of differences in the number of cells between the AD and normal groups (Figure 1D). The proportion of macrophages was significantly higher in the aorta tissues from the AD group (24.51%) compared to those from the normal group (13.69%) (Figure 1D). The proportions of endothelial cells (ECs), fibroblasts, and mesenchymal cells were lower in the AD group than in the normal group (Figure 1D). The numbers and proportions of various cell types in the AD group were as follows: ECs (3180, 5.72%), smooth muscle cells (SMCs) (9958, 17.90%), fibroblasts (6076, 10.92%), mesenchymal cells (1322, 2.38%), macrophages (13,634, 24.51%), T cells (13,715, 24.65%), B lymphocytes (934, 1.68%), monocytes (6461, 11.61%), mast cells (107, 0.19%), and plasmacytes (247, 0.44%). The numbers and proportions of the various cell types in the normal group were as follows: ECs (2034, 8.51%), SMCs (3885, 16.25%), fibroblasts (3857, 16.13%), mesenchymal cells (1124, 4.70%), macrophages (3274, 13.69%), T cells (6078, 25.42%), B lymphocytes (143, 0.60%), monocytes (3200, 13.38%), mast cells (144, 0.60%), and plasmacytes (171, 0.72%) (Table S2).

The top 10 differentially expressed genes (DEGs) in different cell clusters are shown in Figure 1E and Table S3. The cell-type-specific canonical marker genes of the 10 major cell clusters are shown in the UMAP plot (Figure 1G). The major aortic cell types were as follows: ECs (specifically expressing PECAM1, ADGRL4, and AQP1), SMCs (specifically expressing PLN, MYH11, and KCNMB1), fibroblasts (specifically expressing DCN, LOX, and LUM), mesenchymal cells (highly expressing IGFBP5 and TM4SF1), macrophages (specifically expressing CD163, S100A9, and LYZ), T cells (specifically expressing CD3D, CCL5, and CD2), B cells (specifically expressing CD79A, CD79B, and MS4A1), monocytes (expressing CD14, C1QA, and C1QB), mast cells (expressing TPSB2, CPA3, and HPGD), and plasmacytes (expressing IGHG4, IGKC, and JCHAIN) (Figure 1F).

### 3.2. Heterogeneity of Macrophage Phenotypes and Functions between the AD and Normal Groups

**Figure 2:**
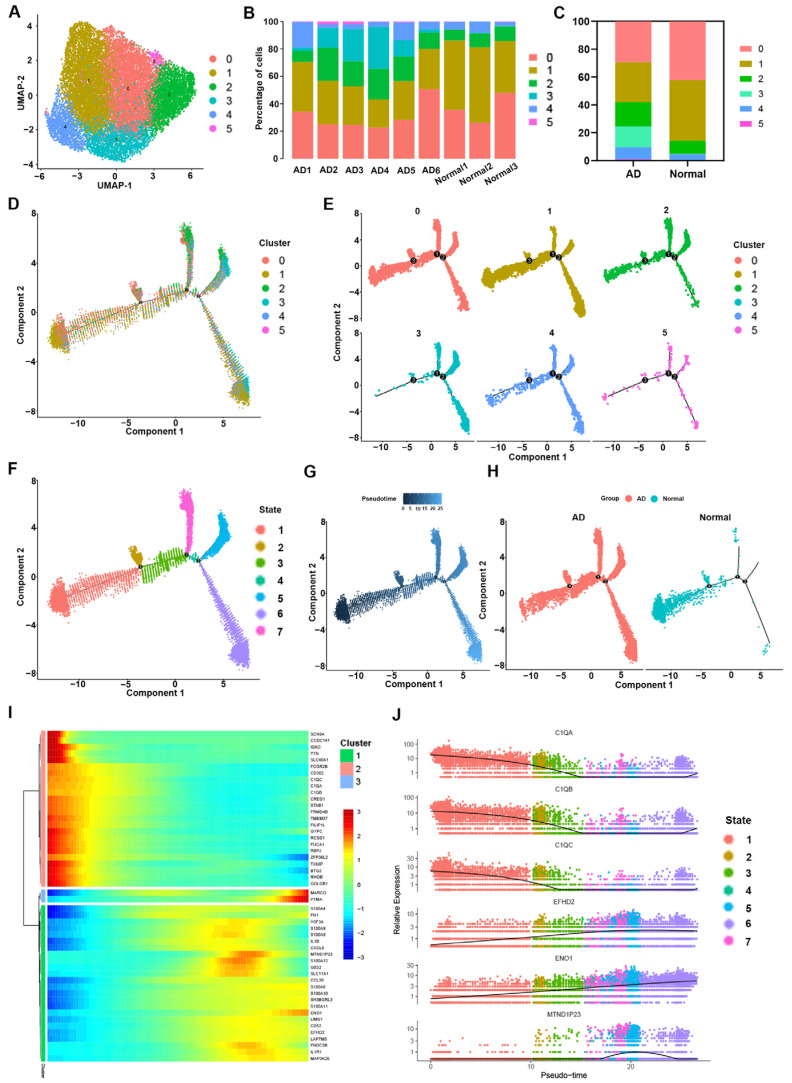
The heterogeneity of macrophages found in AD vs. normal aorta tissue. (**A**) UMAP plot of macrophages reclustered into six subclusters. (**B**) Bar plots of the proportion of macrophage subclusters in nine aortic samples. (**C**) Bar plots of the proportion of macrophage subclusters in the AD and normal groups. (**D**) The Monocle pseudotime trajectory plot shows the progression of six macrophage subclusters. (**E**) Monocle pseudotime trajectory plots of each macrophage subcluster. (**F**) The macrophage subcluster trajectory was separated into seven cell states. (**G**) Monocle pseudotime trajectory plot of macrophages presenting the beginning and end pseudotime profiles. (**H**) The macrophages trajectories in the AD and normal groups. (**I**) The heatmap shows the expression levels of genes grouped into three gene modules according to the pseudotime axis. (**J**) Pseudotime kinetics of the top six genes among the six macrophage subclusters. Each dot represents a cell, different colors represent different clusters, and the ordinate represents the expression level of each gene.

We observed disease-related changes in the macrophages based on the subpopulation and pseudotime trajectory analysis. The aortic macrophages detected in this study (n = 16,908) were further classified into six subclusters after a dimensionality reduction (Figure 2A). The differentially expressed genes in each cluster were sorted by *p*-values and are shown in Table S4. The traditional markers of the M1 and M2 activation states may not be mutually exclusive, according to recent data [22,23]. In addition, several conventional markers are not sufficiently sensitive to identify macrophage programing states in vivo. Therefore, the delineation of unsupervised class algorithms based on a large number of genes for macrophages may be of practical guidance. The six macrophage subclusters displayed different expression patterns (Supplementary Figure S1A). Macrophage subcluster zero (CD74high) expressed high levels of genes involved in antigen presentation, such as the CD74 and MHC II genes (HLA-DRA, HLA-DPA1, and HLA-DPB1). The tissue-resident macrophage marker gene LYVE1 was also expressed in cluster zero [24]. Accordingly, the molecular signature of subcluster zero was enriched for GO terms such as “MHC class II protein complex” and “antigen processing and presentation” (Supplementary Figure S1B). Moreover, KEGG pathway enrichment was mainly associated with antigen presentation and autoimmune diseases (Supplementary Figure S1C). Subcluster one expressed genes related to complementary systems including C1QA, C1QB, and C1QC (Supplementary Figure S1A). In addition, The M2-polarized macrophage marker CD163 was also highly expressed in subcluster one [25] (Table S4). GO and KEGG enriched annotation of subgroup one was mainly in the “extracellular exosome” and “complement and coagulation cascades” (Supplementary Figure S1D,E). Subcluster two expressed high levels of genes involved in the regulation of a number of cellular processes, such as cell cycle progression and differentiation, including S100A8, S100A9, S100A12, and AREG (Supplementary Figure S1A). GO and KEGG enrichment information related to cell proliferation and transcription is shown in Supplementary Figure 1F,G. Subcluster three expressed high levels of pro-inflammatory chemokines (CXCL5, CXCL8, CCL7, and PPBP), thus representing tissue-infiltrated pro-inflammatory macrophages (Supplementary Figure S1A). GO and KEGG terms were mainly associated with chemokine activity and the IL-17 signaling pathway (Supplementary Figure S1H,I). Subcluster four expressed high levels of genes involved in lipid and cholesterol metabolism, such as APOC1, APOE, LPL, FABP4, and FABP5 (Supplementary Figure S1A). The corresponding GO and KEGG enrichment information is shown in Supplementary Figure 1J,K. For the smallest cluster, subcluster five, genes related to the interferon-mediated signaling pathway and immune response, including IFI6, IFI44L, IFIT2, and XAF1, were expressed (Supplementary Figure S1A,L,M).

The percentages of the six macrophage subpopulations in each sample are shown in Figure 2B (Table S5). The trends of the different macrophage subpopulations were more clearly shown in the AD and normal groups (Figure 2C). Macrophage subpopulations zero and one, associated with the antigen presentation and complement systems, respectively, constituted the major components of macrophages in both the AD and normal groups. However, the proportions of both macrophage subpopulations zero and one were significantly lower in the AD group compared to the normal group (Figure 2C). In contrast, the ratios of macrophage subpopulations two, three, four, and five were significantly higher in the AD group (Figure 2C). Notably, the concentration of pro-inflammatory macrophages (subcluster three) was significantly increased in the AD group (from 0.18% in the normal group to 14.96% in the AD group.) (Table S6). In general, the AD group showed increased proportions of macrophages associated with the cell cycle (subcluster two), pro-inflammatory response (subcluster three), and lipid metabolism (subcluster four), and the macrophages displayed a functionally enriched and active state.

To further investigate the role of each macrophage subcluster in AD, we simulated the differentiation trajectories of macrophages using pseudotime methods. The pseudotime analysis distribution positions of the six macrophage subclusters are shown in Figure 2D,E. Seven macrophage states were subsequently identified based on different time nodes (Figure 2F). Based on the schematic diagram of the pseudotime sequence provided by the Monocle2 algorithm, the branch of state one was used as the starting point (Figure 2G). In the pseudotime analysis results of AD tissues, macrophages of the seven states were found to be evenly distributed in different pseudotime sequence locations (Figure 2H). Compared with the AD group, the macrophages in the normal group were mainly distributed around states zero and one, and fewer macrophages were distributed in states three, four, five, six, and seven on the right side of node three (Figure 2H). According to the sequence results of the pseudotime analysis (Figure 2G), the macrophages with a missing distribution in the normal group were mostly functional cells at the end of differentiation (Figure 2H), such as macrophage subcluster three (pro-inflammatory macrophages), subcluster four (lipid metabolism macrophages), and subcluster five (interferon-mediated related macrophages) (Figure 2E). These findings are consistent with the results obtained for the proportional distribution of macrophage subclusters in the normal group shown in Figure 2C. The genes showing significant differences in expression along the pseudotime axis in the macrophages were then clustered into three modules based on their expression patterns in a heatmap (Figure 2I). Module one (green) included genes whose expression level increased initially but subsequently decreased along the pseudotime axis; module two (pink) was composed of genes whose expression level decreased along the pseudotime axis; and module three (blue) was composed of genes whose expression level increased along the pseudotime axis (Figure 2I). The pseudotime kinetics of the top six genes in the seven macrophage states (Figure 2J), namely, C1QA (module two), C1QB (module two), C1AC (module two), EFHD2 (module one), EBO1 (module one), and MTND1P23 (module one) were consistent with the results of the heat map (Figure 2I).

### 3.3. Transcriptome Differences between Macrophages in the AD Group vs. the Normal Group

**Figure 3:**
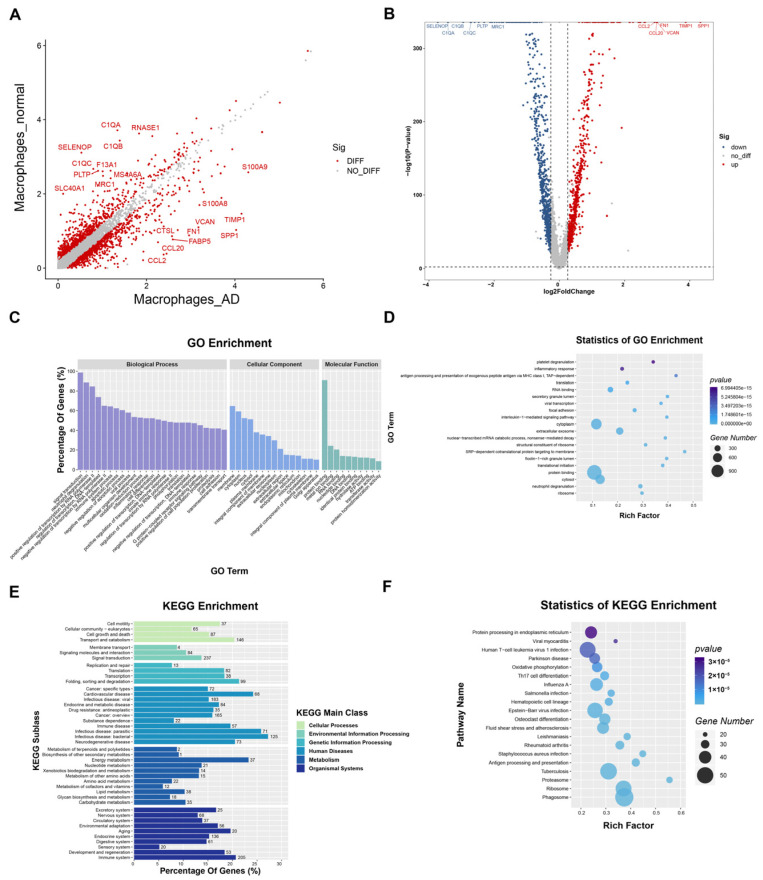
Distribution characteristics of differential genes in macrophages from the AD and normal groups. (**A**) Scatter plot of differential genes in macrophages between the AD group and the normal group. (**B**) Volcano plot of differential genes in macrophages for the AD group vs. the normal group. (**C**) GO enrichment analysis of DEGs. (**D**) Statistical bubble plot of GO enrichment analysis entries. The abscissa is the degree of enrichment, the ordinate is the GO entry, the size of the bubble represents the number of genes, and the color represents the *p*-value. (**E**) KEGG enrichment analysis of DEGs. (**F**) Statistical bubble plot of the KEGG enrichment analysis entries. The abscissa is the degree of enrichment, the ordinate is the KEGG entry, the size of the bubble represents the number of genes, and the color represents the *p*-value.

To gain insight into the role of macrophages in the development of AD, we investigated the biological characteristics of differential genes between the AD and normal groups. Differential genes were obtained by differential analysis with a moderated F-test by using the R package EdgeR (Table S7) [2]. The scatter plot of differential genes in macrophages in the AD and normal groups demonstrates the top 10 up- and downregulated genes as measured by FDR values (Figure 3A, Table S8). The FDR was adjusted by using the Benjamini–Hochberg correction. Among the top 10 upregulated genes in macrophages in the AD group, chemokine- and inflammation-related genes, such as CCL2 (C-C Motif Chemokine Ligand 2) [26], CCL20 [27], S100A8 (S100 Calcium Binding Protein A8) [28], and S100A9 [29], and extracellular matrix and metalloproteinase-related genes, such as VCAN (Versican, the protein encoded is a major component of the extracellular matrix) and TIMP1 (TIMP Metallopeptidase Inhibitor 1), were highly expressed [30,31]. This is consistent with the results of the previous cluster analysis carried out in this study. The top 10 differentially expressed genes in macrophages from the normal group were mainly associated with the complement and immune system, such as C1QA (complement C1q A Chain), C1QB, and C1QC, and the phagocytosis of macrophages, such as MRC1 (Mannose Receptor C-Type 1) [32,33,34]. The results of the volcano plot confirmed the specificity of these genes (Figure 3B, Table S9).

The GO and KEGG enrichment analyses revealed the functions of the differential genes (Figure 3C–F). Among the biological process terms, most of the differential genes were found to be associated with neutrophil degranulation, positive regulation of transcription by RNA polymerase 2, the regulation of transcription, and immune system processes (Figure 3C). Additionally, the molecular function of metal ion binding was consistent with the analysis results for highly expressed genes in macrophages from the AD group (Figure 3C). In terms of the statistical results of the GO analysis, genes associated with antigen processing and presentation, the interleukin-1-mediated signaling pathway, and the degradation of extracellular matrix were found to be highly expressed (Figure 3D). In the KEGG analysis results, the most enriched items included genes associated with transport and catabolism, signaling transduction, gene degradation, energy metabolism, immune system, and cardiovascular disease (Figure 3E,F).

### 3.4. Cell-to-Cell Communication between Macrophages and Other Cells in AD tissue

To further investigate the effect of macrophages on other cells in aortic tissue during AD development, we performed a cell-to-cell communication analysis by tracing the ligand–receptor interactions with our scRNA-seq data.

**Figure 4:**
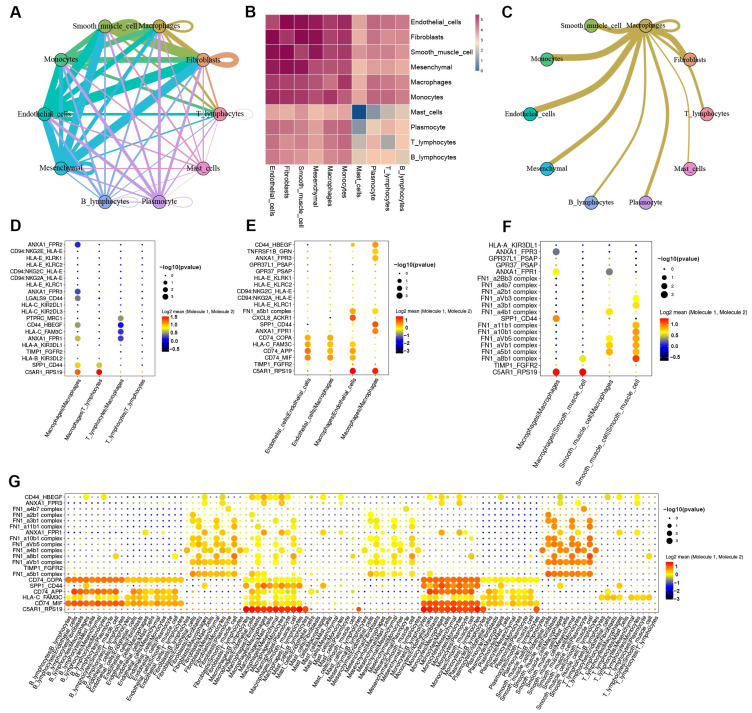
Cell–cell communication between macrophages and other cells in the AD group. (**A**) Network diagram of the weight and number of receptor–ligand interactions between the main cells. (**B**) Heatmap of the strength of communication between major cells. (**C**) Network diagram of communication between macrophages and other cells. (**D**–**F**) Bubble plot of receptor–ligand pairs for communication between macrophages and T cells and endothelial cells and smooth muscle cells, respectively. (**G**) Bubble plot of the receptor–ligand pairs for the 10 major cell types.

The ligand–receptor interactions between macrophages and other major cells were found to be stronger in AD tissue than in normal tissue (Figure 4A–C and Supplementary Figure S2A–C). We found that the communication of macrophages with T cells, endothelial cells, and smooth muscle cells in AD tissues was mainly related to the enhanced binding of C5AR1 (complement C5a receptor 1) to RPS19 (Ribosomal Protein S19) (Figure 4D–F). C5AR1-mediated pro-inflammatory and chemotactic actions of the complement anaphylatoxin C5a may be a pathway by which macrophages affect T cells, endothelial cells, and smooth muscle cells in AD tissues [35,36]. Peng et al. found that C5AR1 deficiency was associated with attenuated deposition of extracellular matrix components (fibronectin and collagen I), reduced cellular infiltrates (CD45 and F4/80), and gene expression of pro-inflammatory and profibrogenic mediators in the kidney [37]. RPS19 (ribosomal protein S19), a component of the 40S small ribosomal subunit, has recently been identified to bind the pro-inflammatory cytokine macrophage MIF (migration inhibitory factor) [38]. The study by Lu et al. concluded that RPS19 is a potent anti-inflammatory agent, which appears to work primarily by inhibiting MIF signaling [38]. Therefore, we hypothesized that the tight binding of ligand receptor pair C5AR1 to RPS19 may suggest a link between the dysfunction of C5AR1 in regulating the extracellular matrix and the loss of the anti-inflammatory effect of RPS19. In addition, the expression of the antigen recognition ligand CD74 secreted by macrophages and the corresponding receptors MIF (Migration Inhibitory Factor), APP (Amyloid Beta Precursor Protein), and COPA (COPI Coat Complex Subunit Alpha) in endothelial cells was significantly higher in AD tissues than in normal tissues (Figure 4E and Supplementary Figure S2E). The upregulation of the ligand–receptor pair CD74-MIF may indicate the recruitment of macrophages by endothelial cells in AD tissues and the initiation of macrophage-mediated inflammatory and immune processes [39,40]. Furthermore, the binding of FN1 (Fibronectin 1, dimeric or multimeric form at the cell surface and in the extracellular matrix) to its receptor was weakened between smooth muscle cells in AD tissue compared to normal tissue (Figure 4F and Supplementary Figure S2F). Signal crosstalk between the 10 major cell types in AD tissues and normal tissues is shown in Figure 4G and Supplementary Figure S4G, respectively (Figure 4G and Supplementary Figure S2G). These results suggest that cellular communication between macrophages, endothelial cells, and smooth muscle cells in AD tissues was increased in terms of inflammatory and immune aspects, thereby promoting the degradation of extracellular-matrix-associated proteins between smooth muscle cells.

### 3.5. Transcriptional Regulation of Macrophages in AD Tissues

**Figure 5:**
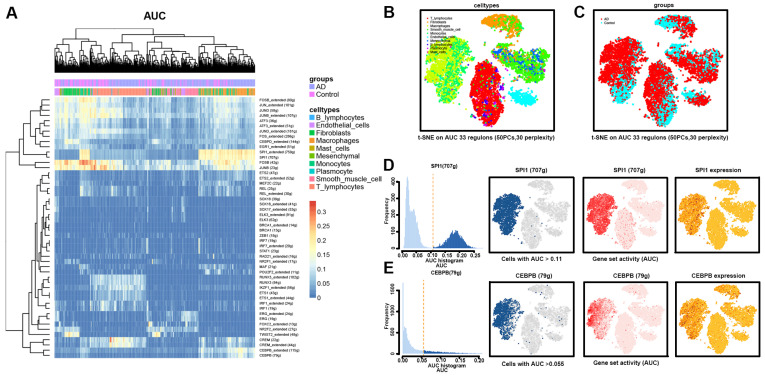
Transcriptional regulation of the 10 main cell types in the AD and normal groups. (**A**) Heatmap indicating expression regulation by TFs analyzed with SCENIC in the 10 main cell types. The numbers between brackets indicate the (extended) regulons for the respective TFs. (**B**) t-SEN plot showing the cell type distribution based on a dimensionality reduction by 33 regulons. (**C**) t-SEN plot showing the sample distribution based on a dimensionality reduction by 33 regulons. (**D**) Characterization of the AUC of the macrophage-associated transcriptional regulon SPI1. TF regulon activities were quantified using the AUC. (**E**) Characterization of the AUC of the macrophage-associated transcriptional regulon CEBPB. TFs, transcription factors; SCENIC, single-cell regulatory network inference and clustering; t-SNE, t-distributed stochastic neighbor embedding.

The SCENIC algorithm was performed to investigate the transcription factors (TFs) that may induce macrophage heterogeneity in AD tissues. We were able to distinguish variations in the activities of regulons among the 10 major cell types using the calculated regulon activity scores (RAS) of transcription factors (Figure 5A). The Spleen Focus Forming Virus Proviral Integration Oncogene (SPI1) and CCAAT Enhancer Binding Protein Beta (CEBPB) regulon activities of macrophages were greater in AD tissues. SPI1, a recognized key TF for the maturation of macrophages [41,42], may be involved in macrophage polarization and pro-inflammatory processes [43]. CEBPB, an important transcription factor that regulates the expression of genes involved in immune and inflammatory responses [44,45,46], is essential for gene expression induction in activated macrophages and plays a major role in immune responses such as CD4(+) T cell responses and granuloma formation [47,48].

The t-SNE dimensionality reduction analysis was performed by cell type and disease grouping based on the AUC values of 33 regulons (Figure 5B,C). The results show a higher concentration of macrophage-associated regulons in AD tissues than in normal tissues. Visualization of the t-SNE plots based on AUC values, gene set activity, and expression showed high specificity of SPI1 and CEBPB in macrophage-associated TFs (Figure 5D,E). The above results imply that the crucial TFs SPI1 and CEBPB may contribute to the development of aortic wall lesions in AD tissue by inducing macrophage polarization and stimulating the pro-inflammatory effects of macrophages.

### 3.6. Histopathological Validation

**Figure 6:**
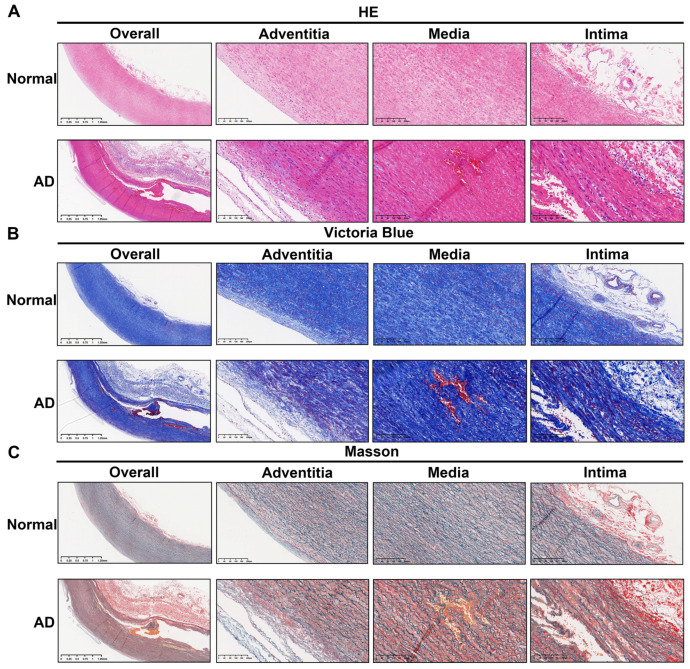
Histopathological differences between AD and normal tissues. (**A**–**C**) HE, Victoria Blue, and Masson’s trichrome staining were used to observe differences in the cellular structure, elastic fibers, and collagen fibers of ascending aorta sections from AD and normal participants. The image on the left shows an overview of the whole ascending aorta wall tissue (magnification: ×20). The three images on the right show magnifications of the adventitia, media, and intima of the ascending aortic wall, respectively (magnification: ×100). HE, hematoxylin–eosin.

The HE, Victoria Blue, and Masson’s trichrome staining results show the overall morphology and the distribution and arrangement of cells, elastic fibers, and collagen fibers in the ascending aorta wall tissue samples from the AD and normal groups (Figure 6A–C). Significant breakage of elastic and collagen fibers in the aortic wall tissue occurred in the AD group (Figure 6A–C). In addition, the intima of the aortic wall tissue was significantly thickened in the AD group, which could represent an increase in secretory units and an ongoing inflammatory response. In the high-resolution images produced by HE staining, smooth muscle cells located in the media in AD tissue were shown to have lost their complete cell morphology and regular arrangement order (Figure 6A).

**Figure 7:**
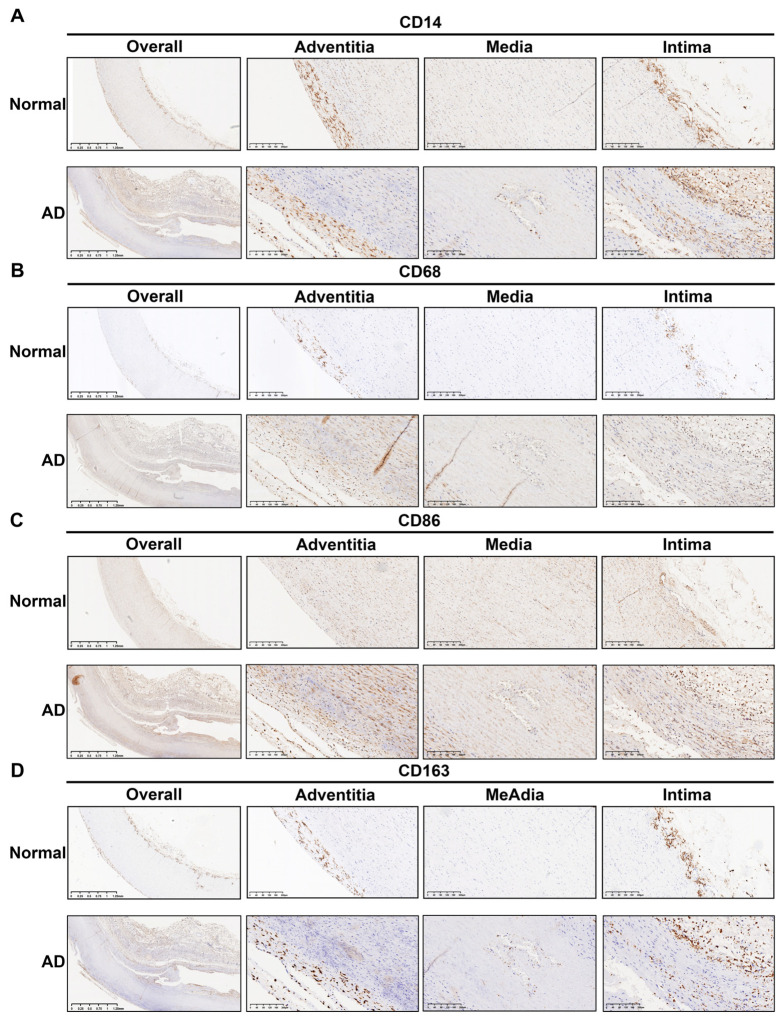
Immunohistochemical analysis of macrophages and monocytes in sections of AD and normal samples. (**A**) CD14 antibody labeling showing the distribution of monocytes in tissues. (**B**) CD68 antibody labeling showing the distribution of macrophages in tissues. (**C**) CD86 antibody labeling showing the distribution of M1 macrophages in tissues. (**D**) CD163 antibody labeling showing the distribution of M2 macrophages in tissues. The image on the left shows an overview of the whole ascending aorta wall tissue (magnification: ×20). The three images on the right show magnifications of the adventitia, media, and intima of the ascending aortic wall, respectively (magnification: ×100).

Immunohistochemical staining for marker genes of monocytes (CD14) and macrophages (CD68, CD86, and CD163) demonstrated the accumulation of macrophages in the aortic wall tissue of the AD group (Figure 7A–D). Based on CD14 staining, the concentration of monocytes was found to be significantly increased in the intima, the tear site of the media, and the adventitia (Figure 7A). The positive staining of macrophages in AD tissues was stronger than that in normal tissues, as seen by the staining of CD68, CD86, and CD163 (Figure 7B–D).

**Figure 8:**
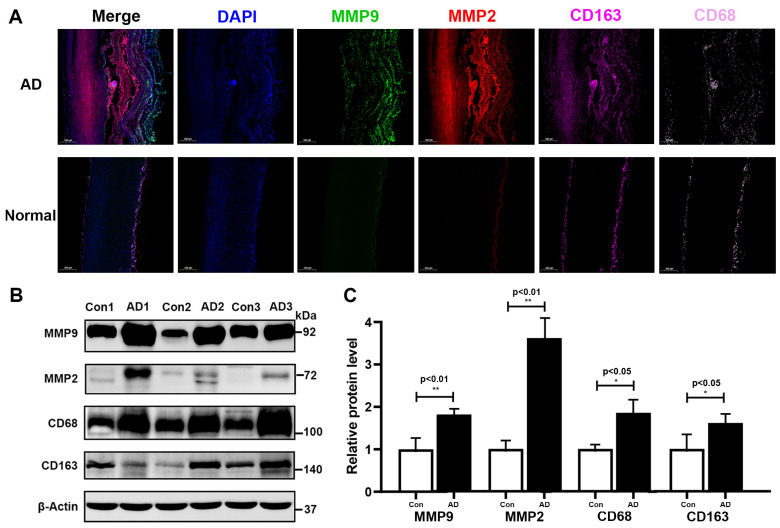
Detection of macrophage markers and metallomatrix proteinases in AD and normal tissues. (**A**) Representative mIF images of normal and dissected human ascending aortas stained with DAPI (blue), MMP9 (green), MMP2 (red), CD163 (magenta), and CD68 (pink) (Magnification: ×20). (**B**) Western blotting experiments showing the protein expression levels of MMP9, MMP2, CD163, and CD68 in AD and normal tissues. (**C**) Bar graph of protein expression in AD tissue relative to normal tissue. * represents *p* < 0.05; ** represents *p* < 0.01. mIF: multiplexed immunofluorescence.

The four-plex mIF assay and protein blotting experiments verified the high expression levels of MMP9, MMP2, CD163, and CD68 in AD tissues (Figure 8A–C). The fluorescence intensity of MMP9 and MMP2 was significantly higher in the ascending aortic wall of AD patients than in normal ascending aortic tissue, and the fluorescence was mainly concentrated in the intima (MMP9) and tear sites (MMP2) of the aortic wall of AD tissue (Figure 8A). The macrophage markers CD163 and CD68 were also expressed more strongly in AD tissues compared to normal tissues (Figure 8A). The protein extracted from the ascending aorta tissue was subjected to Western blot analysis, and it was once again found that the expression levels of MMP9, MMP2, CD163, and CD68 were higher in AD tissue than in normal aortic tissue (Figure 8B,C).

## 4. Discussion

ATAAD is a type of aortic wall lesion involving tissue injury, repair, and remodeling using inflammatory cells [49]. Macrophage infiltration has been found in the aortic walls of patients with aortic dissection, but the contribution of these cells to disease progression has not been elucidated [5,7]. In this study, we investigated the role of macrophages in the pathogenesis of aortic dissection through single-cell sequencing of ascending aortic walls from ATAAD patients and normal heart transplant donors. We found that the concentration of pro-inflammatory macrophages was significantly increased in AD tissues. Moreover, a differential expression analysis of the macrophage transcriptome in AD tissues vs. normal tissues confirmed the high expression of chemokines (CCL2, CCL20, and S100A8) in AD tissues. The cellular communication between macrophages and smooth muscle cells in terms of fibronectin (FN1) was found to be higher in AD tissue than in normal tissue. A differential analysis of transcription factors revealed that transcription factors associated with FN1, which constitutes the extracellular matrix, were significantly overexpressed in the macrophages of AD tissue. MMPs are enzymes known to remodel extracellular matrix components, including FN1 [50]. The high expression of MMPs in AD tissues was confirmed by immunofluorescence and Western blotting analyses. Therefore, this study concludes that the high expression of pro-inflammatory macrophage-mediated MMPs may contribute to the decomposition and tearing of the aortic wall media in ATAAD patients.

The elevated proportion of macrophages in the aortic walls of ATAAD patients is an important finding of this study. With the continuous development of single-cell research in aortic dissection, the role of macrophages in AD is receiving increased attention [51]. Liu et al. identified an increased number of macrophages in AD tissue by single-cell sequencing of the aortic walls of three patients with type A aortic coarctation and two heart transplant donors and suggested that circulating macrophages were involved in infiltration [7]. In the single-cell sequencing results of thoracic aortic aneurysms similar to aortic dissection, activated macrophages were also found to be increased in concentration, and chemokine-related genes were highly expressed [12]. Compared to an aortic aneurysm, AD may involve more significant macrophage infiltration [52]. In a mouse model of aortic disease, intervention exposure induced significant expansion of the total macrophage population compared with a sham group, and the expansion continued during abdominal aortic aneurysm progression [53]. However, few studies have the analyzed detailed changes in different macrophage subsets. In this study, macrophages in AD tissues were found to be richly heterogeneous (Figure 2C). Moreover, the proportions of pro-inflammatory macrophages (subcluster three), interferon-mediated macrophages (subcluster four), and lipid-metabolism-related macrophages (subcluster five) were higher in AD tissues than in normal tissues. Recent studies have revealed that inflammation plays a significant role in the progression of AD and that macrophages are the primary source of inflammation in the aortic wall [54,55]. In the pseudotime analysis, the complement-related genes C1QA, C1QB, and C1QC emerged as key genes for delineating the evolution of macrophage subpopulations (Figure 2I,J). This finding may indicate that inflammatory responses involving macrophages are mediated by the complement system. Therefore, the pro-inflammatory macrophages and interferon-mediated macrophages identified in this study may be involved in the inflammatory process in AD tissues. The increase in lipid-metabolism-related macrophages may be associated with the progression of inflammation-associated aortic atherosclerosis [24].

The increased chemokine expression identified in macrophages of AD tissues may be associated with aortic wall inflammation. In this study, the expression levels of the classical chemokines CCL2 and CCL20, as well as those of S100A8 and S100A9, which are indirectly involved in inflammation, were significantly higher in macrophages from AD tissues than in those from normal tissues (Figure 3A). It was found that resident macrophages produce CCL2, which induces the recruitment of inflammatory monocytes (iMos) into the aortic root, the first step required to trigger aortitis [56,57]. Then, iMos differentiates into monocyte-derived dendritic cells and produces IL-1β via the Dectin-2/NF-κB/NAPL3 inflammasome pathway, leading to amplified neutrophil and iMo recruitment by chemokine production in inflamed vessels [56]. In addition, the pro-inflammatory effect of CCL2 in macrophages from AD tissues was confirmed through both single-cell sequencing and bulk sequencing results [12,58]. Wang et al. found that S100A8/9 was highly expressed in a mouse model of spontaneous aortic inflammation [59]. Urokinase plasminogen activator-overexpressing macrophages were found to secrete large amounts of S100A8/9, which, in turn, stimulated atherosclerosis and aortic dilation [60]. Ganta et al. found that S100A8/S100A9, as downstream regulators of vascular endothelial growth factor receptor 1, may induce an M1-like phenotype through a S100A8/S100A9-mediated calcium influx [61]. M1 macrophages are mainly involved in pro-inflammatory responses as classically activated macrophages [62]. Notably, genes involved in extracellular matrix (ECM) repair rather than degradation, especially VCAN, FN1, and TIMP1, were found to be significantly expressed in the macrophages of AD tissues (Figure 3A). Liu et al. also found high expression of similar genes (VCAN, TIMP1, EREG, and AREG) in macrophages by single-cell sequencing of three type A aortic dissection cases [7]. The results of the scRNA-seq study of ascending thoracic aortic aneurysm by Li et al. also showed high expression of inflammatory factors and tissue repair cytokines, including VCAN, TIMP1, AREG, and EREG [12]. Therefore, the pro-inflammatory and pro-extracellular matrix repair effects of AD tissue-derived macrophages may be stronger than those of macrophages from normal aortic tissue.

Macrophages abrogate the structural stability of the aortic wall by communicating with T cells and the endothelial cells and smooth muscle cells (SMC) that make up the aortic wall [63]. In this study, the cell-to-cell communication of macrophages with T cells, endothelial cells, and SMCs was mainly through ligand–receptor pairs, such as C5AR1 to RPS19 and FN1 to its receptor (Figure 4). The intracellularly active complement is a vital orchestrator of cellular metabolic events underlying T cell effector responses [64,65]. Data from Niyonzima et al. showed the unexpected intracellular formation of C5 translocase and identified C5AR1 as a direct regulator of the inflammatory output from macrophages [64]. Previous studies have shown that T cells contribute to macrophage recruitment and the secretion of inflammatory factors, such as TNF-α, by secreting various interleukins, such as IL32 and IL17 [66,67]. Furthermore, some negative regulators of T cells hamper macrophage recruitment and CD8+ T cell-mediated macrophage death while limiting the activation of CD8+ T cells [68]. Cellular communication between macrophages and endothelial cells is mainly mediated by the ligand–receptor pairs CD74-MIF, CD74-APP, and CD74-COPA (Figure 3E). Endothelial cells in AD tissues may recruit circulating monocytes/macrophages through these ligand–receptor pairs [10,69]. In addition, Poddar et al. found that L-homocysteine may contribute to the initiation and progression of vascular disease by altering endothelial cell function through upregulation of MCP-1 and IL-8 expression and secretion, which in turn promotes monocytes/macrophages recruitment [70]. Inflammatory and immune responses due to endothelial cell damage or dysfunction also promote the recruitment of monocytes/macrophages, which in turn produce aortic lesions, including vascular SMCs [71]. The degeneration and death of SMCs, a major component of the media, contributes to the development of aortic dissection [72]. In this study, the reduced crosstalk between the SMCs of AD tissue via FN1 and its receptor may indicate a decrease in SMC activity (Figure 4F and Supplementary Figure S2F). The complex inflammatory and immune microenvironment caused by macrophages and cytotoxic T cells contributes to the aortic SMC phenotype switch and the loss of original function [11,73]. Previous research has shown that phenotype-switched SMCs progressively lose their contractile and synthetic functions, while their functions of proteolysis, endocytosis, phagocytosis, and lysosomal clearance of extracellular matrix and apoptotic cells are enhanced [74]. Intriguingly, SMCs in inflammatory settings can acquire a limited repertoire of macrophage markers and functions through the biosynthesis of degraded organelles in the mTOR/β-catenin/MITF-dependent pathway but are distinguished from conventional macrophages by the lack of hematopoietic lineage markers and certain immune effectors [74]. Hu et al. found that IL-18 may promote the differentiation of M1 macrophages and increase the apoptosis of SMCs induced by macrophages [75]. Therefore, the inflammatory environment constructed by macrophages makes SMCs dysfunctional and leads to the degradation of elastic fibers, resulting in aortic wall medial lesions.

Through a transcriptional regulation analysis, the high expression of the TFs SPI1 and CEBPB in the macrophages of AD tissues was associated with inflammation and immune regulation (Figure 5A). Single-cell RNA-seq studies by Cui et al. identified SPI1 as a key regulon and potential immunotherapeutic target for macrophage maturation and polarization [43]. Some findings suggest that high SPI1 expression may prevent macrophage migration [76]. Determination of the specific functions of SPI1 in the regulation of macrophages and immune processes requires more research [77]. CEBPB is a transcription regulon that influences the direction of macrophage polarization [78]. Previous studies have shown that CEBPB is highly expressed in pro-inflammatory M1 macrophages, while its expression is low in anti-inflammatory M2 macrophages [79,80]. Particularly, CEBPB knockdown was found to decrease the expression of M1-type markers (iNOS) while increasing the expression of M2-type markers (FIZZ1, Ym1, and ARG1) [79]. Additionally, the silencing of CEBPB was found to trigger anti-inflammatory M2-like polarization and inhibit foam cell formation in a mouse model of atherosclerosis [79].

MMPs secreted by macrophages play an important role in the destruction of the AD tissue structure. In this study, the expression levels of MMP9 and MMP2 in AD tissues were confirmed to be higher than those in normal aortic tissues by immunofluorescence and Western blot analyses (Figure 8A,B). Additionally, the macrophage markers CD68 and CD163 were found to be highly expressed in AD tissues (Figure 8B). The results of the scRNA-seq study conducted by Liu et al. in immune cells of AD are consistent with the results of our study [7]. The regulation of MMPs by macrophages in AD tissues deserves further exploration. Li et al. found that the infusion of monocytes into a mouse model of AD resulted in an increase in aortic rupture [81]. Conversely, targeted depletion of monocytes/macrophages inhibited the infiltration of macrophages in the aortic wall and the occurrence of AD. A further quantitative mass spectrometry analysis revealed significant differences in the intensity of multiple MMPs following monocyte/macrophage depletion. As verified by immunofluorescence, macrophage infiltration significantly upregulated MMP9 and MMP9 co-localized with macrophages in the aortic tear area [81]. Our study also identified synergistic enhancement of macrophages and MMP9 in AD tissues (Figure 8A). High expression of MMPs in AD tissues has been found in both mouse models and clinical patients. In a mouse model of aortic aneurysm/dissection, the lesioned aortic tissue was accompanied by elastic lamina degradation, increased macrophage infiltration, and elevated expression of MMP9 and MMP2 [82,83]. Liao et al. analyzed blood samples from 30 acute AD patients and 30 age- and sex-matched healthy controls and found higher rates of transcription and secretion of MMP-1, MMP-2, MMP-3, MMP-8, MMP-9, and MMP-12 by monocytes/macrophages from acute AD patients than in those from healthy controls [84]. Barhoumi et al. found that MMP2 knockout in immune cells prevented angiotensin II-induced vascular injury in bone marrow transplantation experiments in mice [85]. In addition, MMPs secreted by macrophages have been shown to play important roles in regulating inflammation, degradation of the extracellular matrix, and tissue remodeling in the aortic wall [86]. Therefore, aortic wall disruption by macrophage-regulated MMPs in AD tissues may be one of the pathways of AD pathogenesis.

In conclusion, we revealed the landscape of macrophages in ATAAD patients at the single-cell level. We found that the pro-inflammatory macrophage subcluster plays a key role in the pathogenesis of ATAAD. Pro-inflammatory macrophages in ATAAD tissue may damage the normal structure and microenvironment of aortic tissue by secreting inflammatory factors and MMPs, causing the formation of ATAAD. In summary, our study provides a preliminary evaluation of the role of macrophages in ATAAD, and the results could aid in the development of therapeutic options in the future.

## Data Availability

Data are contained within the article or supplementary material. Additional data that support the findings of this study are available from the corresponding author upon reasonable request. The data presented in this study have been uploaded on the GEO database. The GEO accession number is GGSE213740.

## Supplementary Materials

The following supporting information can be downloaded at: https://www.mdpi.com/article/10.3390/biom13020399/s1. Figure S1: Transcriptome distribution characteristics of each macrophage subcluster; Figure S2: Cell–cell communication between macrophages and other cells in the normal group; Table S1: Patient information for ascending aortic samples (n = 9); Table S2: Number and proportion of each cell; Table S3: Top 10 DEGs among the 10 major clusters defined in ascending aorta tissue; Table S4: Marker gene of macrophage subclusters; Table S5: Number and proportion of macrophages subclusters; Table S6: Number and proportion of macrophage subclusters in AD and normal groups; Table S7: Differentially expressed genes between macrophages in AD group and normal group; Table S8: Differentially expressed gene characteristics between macrophages in AD group and normal group.
