# Supplementary material for: Single-Cell RNA-Seq Analysis Reveals Macrophages Are Involved in the Pathogenesis of Human Sporadic Acute Type A Aortic Dissection
Source: Biomolecules. 2023 Feb 20;13(2):399. doi: 10.3390/biom13020399 (PMC9952989; doi:10.3390/biom13020399)
Supplement: Supplementary file 1 [file biomolecules-13-00399-s001.zip › Supplementary Material.pdf]

Supplementary Material

1 Supplementary Figures and tables

1.1 Supplementary Figures S1, S2

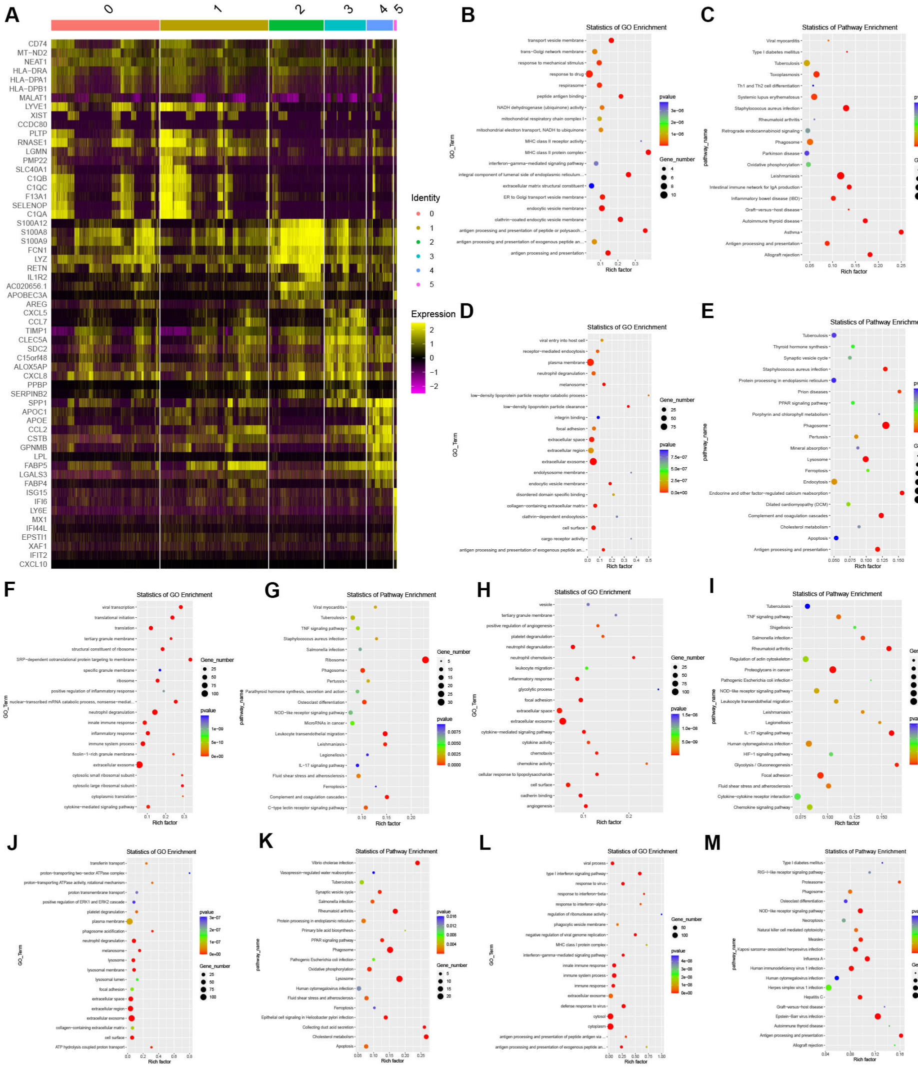

Supplementary Figure S1 | Transcriptome distribution characteristics of each macrophage subclusters. **(A)**Heatmap showing distinct expression profiles of the six macrophage subclusters. **(B, C)** GO and KEGG terms enriched in the molecular signatures of subcluster 0. **(D, E)** GO and KEGG terms enriched in the molecular signatures of subcluster 1. **(F, G)** GO and KEGG terms enriched in the molecular signatures of subcluster 2. **(H, I)** GO and KEGG terms enriched in the molecular signatures of subcluster 3. **(J, K)** GO and KEGG terms enriched in the molecular signatures of subcluster 4. **(L, M)** GO and KEGG terms enriched in the molecular signatures of subcluster 5. Adjusted P value of hypergeometric test <0.05.

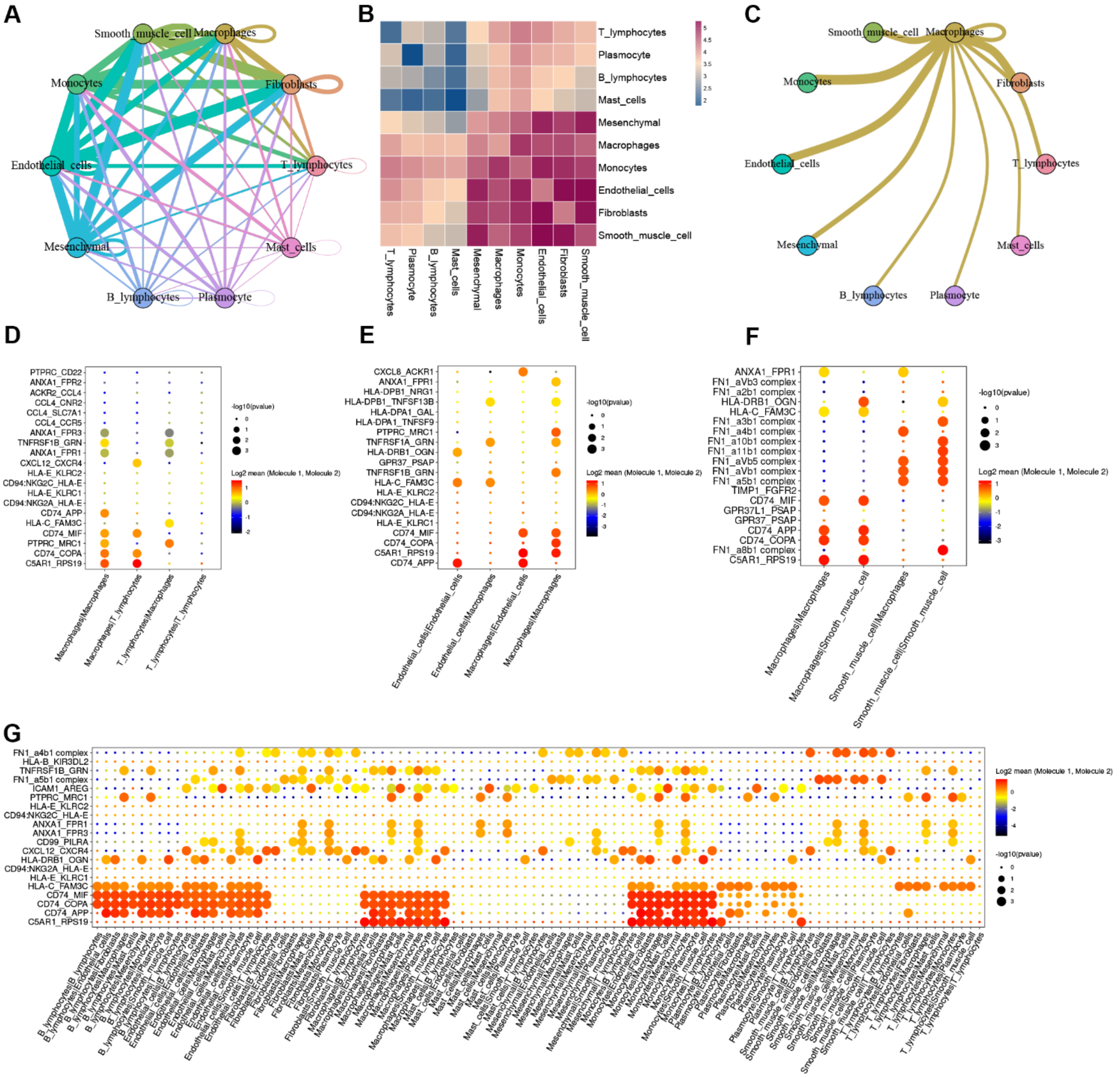

Supplementary Figure S2 | Cell–cell communication between macrophages and other cells in the normal group. **(A)** Network diagram of the weight and number of receptor - ligand interactions between main cells. **(B)** Heat map of the strength of communication between major cells. **(C)** Network diagram of communication between macrophages and other cells. **(D, E, F)** Bubble plot of receptor-ligand pairs for communication between macrophages and T cells, endothelial cells and smooth muscle cells, respectively. **(G)** Bubble plot of the receptor-ligand pairs between the 10 major cells.

1.2 Supplementary Tables

Table S1. Patient Information for Ascending Aortic Samples (n = 9).

Table S2. Number and proportion of each cell.

Table S3. Top 10 DEGs among the 10 major clusters defined in ascending aorta tissue.

Table S4. Marker gene of macrophage subclusters.

Table S5. Number and proportion of macrophages subclusters.

Table S6. Number and proportion of macrophage subclusters in AD and normal groups.

Table S7. Differentially expressed genes between macrophages in AD group and normal group.

Table S8. Differentially expressed gene characteristics between macrophages in AD group and normal group.
