# Supplementary material for: Single-Cell RNA-Seq Analysis Reveals Macrophages Are Involved in the Pathogenesis of Human Sporadic Acute Type A Aortic Dissection
Source: Biomolecules. 2023 Feb 20;13(2):399. doi: 10.3390/biom13020399 (PMC9952989; doi:10.3390/biom13020399)
Supplement: Supplementary file 1 [file biomolecules-13-00399-s001.zip › Table S1.pdf]

TABLE S1 Patient Information for Ascending Aortic Samples (n = 9).

Variable	AD1	AD2	AD3	AD4	AD5	AD6	Normal1	Normal2	Normal3
Gender	M	F	F	M	M	F	M	M	M
Age (year)	59	64	25	58	50	38	58	57	58
Ethnicity	Chinese	Chinese	Chinese	Chinese	Chinese	Chinese	Chinese	Chinese	Chinese
Race	Asian	Asian	Asian	Asian	Asian	Asian	Asian	Asian	Asian
Diagnosis and comments	ATAAD	ATAAD	ATAAD	ATAAD	ATAAD	ATAAD	Heart transplant donor	Heart transplant donor	Heart transplant donor
Smoking status	Yes	No	No	No	Yes	No	No	Yes	No
Diabetes mellitus	No	No	No	Yes	No	No	No	No	No
Hypertension	Yes	Yes	No	Yes	Yes	Yes	Yes	No	Yes
Chronic obstructive pulmonary disease	No	No	No	No	No	No	No	No	No
Aortic value regurgitation	No	Yes	No	Yes	No	No	No	No	No
Bicuspid aortic value	No	No	No	No	No	No	No	No	No
Reoperation	No	No	No	No	No	No	No	No	No

F, female; M, male; NA, not available; and ATAAD, acute type A aortic dissection.
