# Supplementary figures and images for: Single-Cell RNA-Seq Analysis Reveals Macrophages Are Involved in the Pathogenesis of Human Sporadic Acute Type A Aortic Dissection
Source: Biomolecules. 2023 Feb 20;13(2):399. doi: 10.3390/biom13020399 (PMC9952989; doi:10.3390/biom13020399)

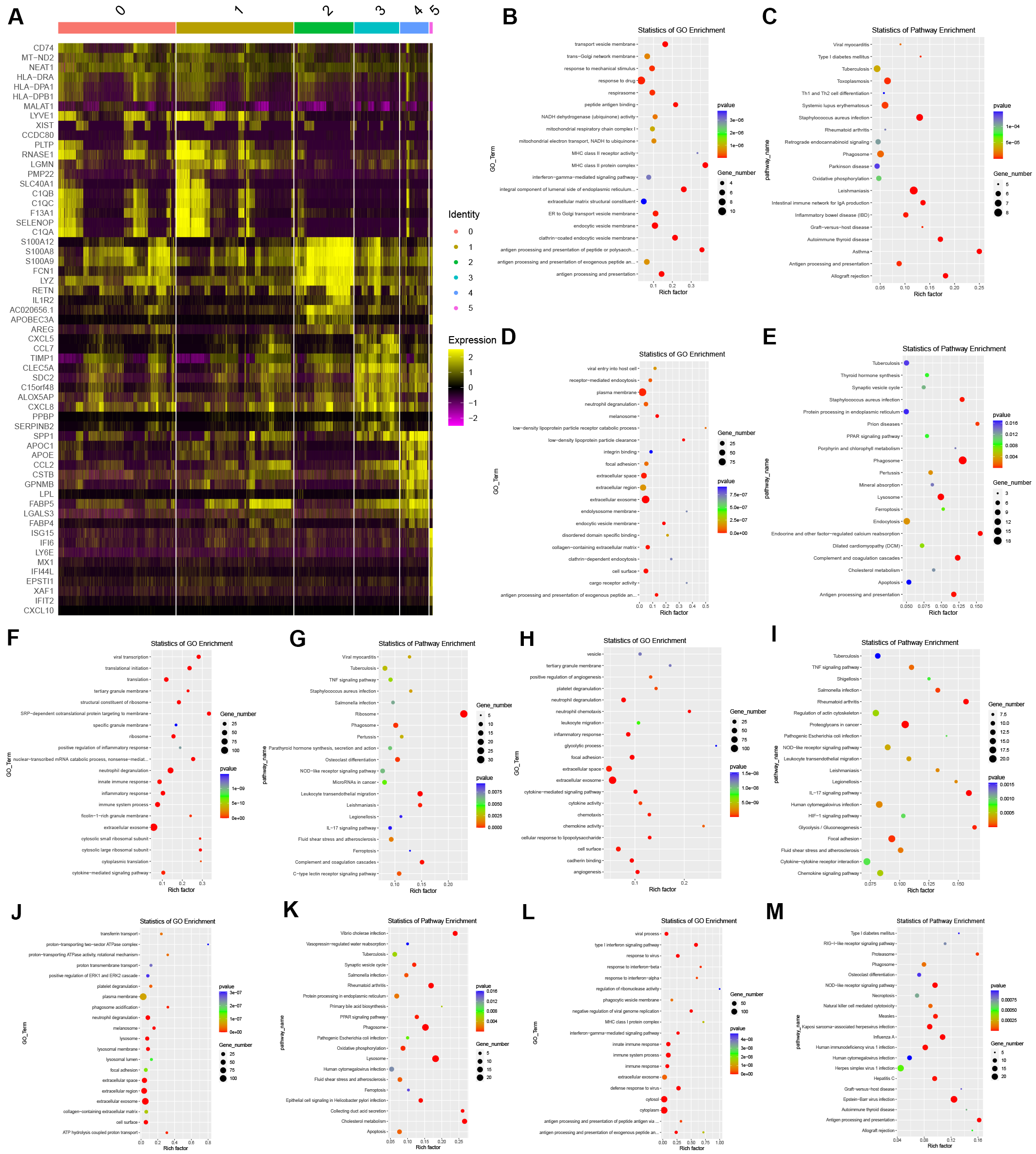

Supplement: Supplementary file 1 [file biomolecules-13-00399-s001.zip › Supplementary Figure S1.tif]

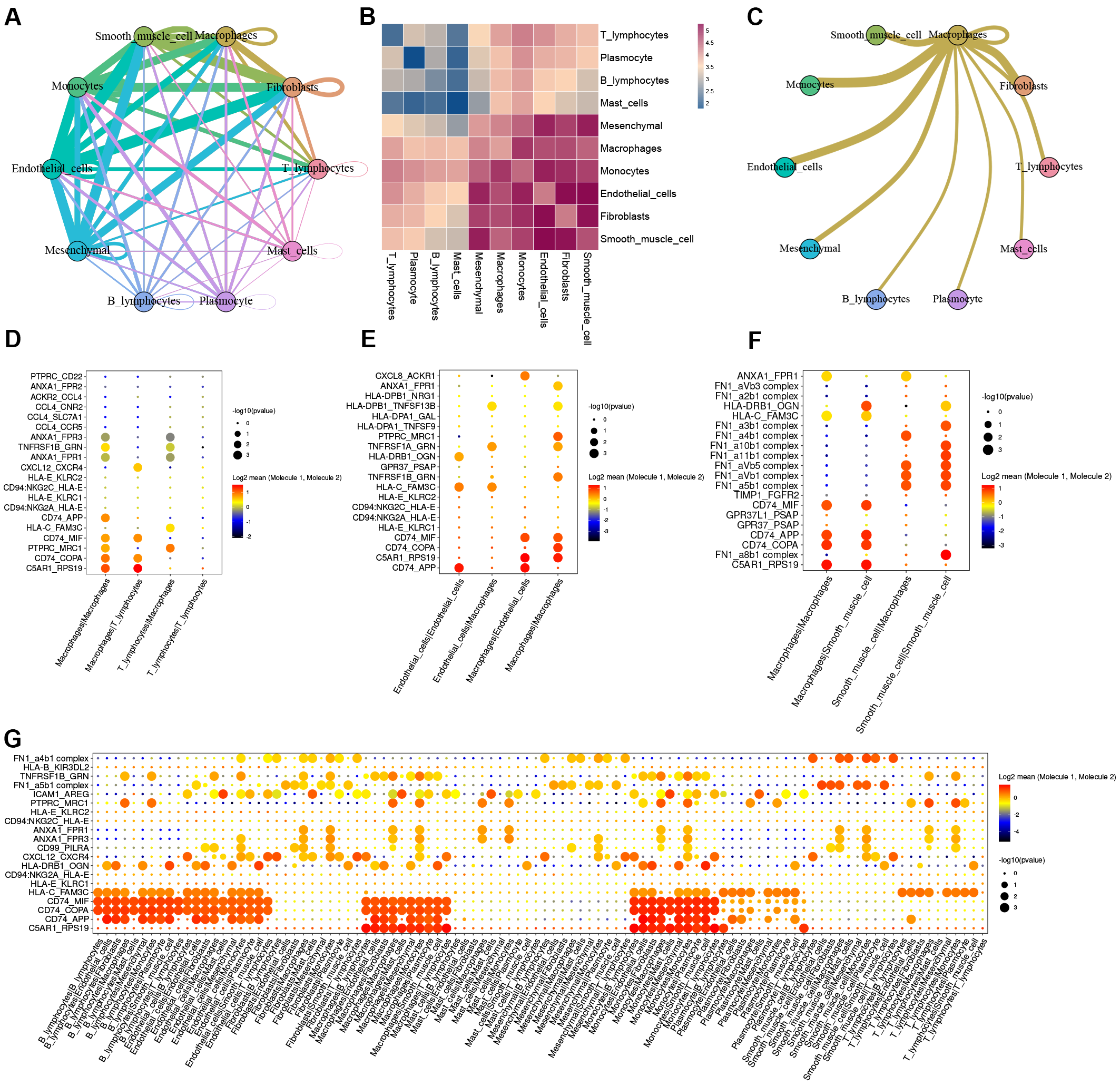

Supplement: Supplementary file 1 [file biomolecules-13-00399-s001.zip › Supplementary Figure S2.tif]
